# Dietary Information Improves Model Performance and Predictive Ability of a Noninvasive Type 2 Diabetes Risk Model

**DOI:** 10.1371/journal.pone.0166206

**Published:** 2016-11-16

**Authors:** Tianshu Han, Shuang Tian, Li Wang, Xi Liang, Hongli Cui, Shanshan Du, Guanqiong Na, Lixin Na, Changhao Sun

**Affiliations:** National Key Discipline, Department of Nutrition and Food Hygiene, School of Public Health, Harbin Medical University, Harbin, P. R. China; University of Rochester, UNITED STATES

## Abstract

There is no diabetes risk model that includes dietary predictors in Asia. We sought to develop a diet-containing noninvasive diabetes risk model in Northern China and to evaluate whether dietary predictors can improve model performance and predictive ability. Cross-sectional data for 9,734 adults aged 20–74 years old were used as the derivation data, and results obtained for a cohort of 4,515 adults with 4.2 years of follow-up were used as the validation data. We used a logistic regression model to develop a diet-containing noninvasive risk model. Akaike’s information criterion (AIC), area under curve (AUC), integrated discrimination improvements (IDI), net classification improvement (NRI) and calibration statistics were calculated to explicitly assess the effect of dietary predictors on a diabetes risk model. A diet-containing type 2 diabetes risk model was developed. The significant dietary predictors including the consumption of staple foods, livestock, eggs, potato, dairy products, fresh fruit and vegetables were included in the risk model. Dietary predictors improved the noninvasive diabetes risk model with a significant increase in the AUC (delta AUC = 0.03, *P*<0.001), an increase in relative IDI (24.6%, *P*-value for IDI <0.001), an increase in NRI (category-free NRI = 0.155, *P*<0.001), an increase in sensitivity of the model with 7.3% and a decrease in AIC (delta AIC = 199.5). The results of the validation data were similar to the derivation data. The calibration of the diet-containing diabetes risk model was better than that of the risk model without dietary predictors in the validation data. Dietary information improves model performance and predictive ability of noninvasive type 2 diabetes risk model based on classic risk factors. Dietary information may be useful for developing a noninvasive diabetes risk model.

## Introduction

A number of noninvasive diabetes risk models have been developed to predict the risk of developing type 2 diabetes and to identify high-risk individuals [1–11]. The advantages of a noninvasive diabetes risk model are its lower cost and greater convenience compared with the blood glucose test. Furthermore, it can be widely used in a population-based screening program for health promotion and disease prevention. Thus far, most of the noninvasive diabetes risk models have been developed for Caucasian populations [1–5]. However, Glümer et al. have evaluated the performance of one diabetes risk model for Caucasians in nine populations of diverse ethnic origin and found that the risk model did not perform well in other ethnic groups [12]. This finding suggested that these diabetes risk models that were developed for Caucasian populations may be limited when applied to an Asian population. However, very few diabetes risk models have been developed for the Asian population [6–11]. Furthermore, most of the risk models developed for Caucasians did not include dietary predictors, and there is no diabetes risk model including dietary predictors for Asians.

Many cohort studies have confirmed that diet plays an important role in the development of type 2 diabetes. Many studies have suggested that type 2 diabetes can be prevented in high-risk individuals by intensive behavioral interventions targeting the diet [13–15]. However, compared with classic risk factors such as age, gender, physical activity, a family history of disease, and anthropometrics indices, previous studies have underestimated the impact of dietary predictors in these established diabetes risk models. In 2007, Simmons et al. reported that dietary predictors did not improve the predictive ability of noninvasive diabetes risk models using classic risk factors and comparing the value of C-statistics between risk models with and without dietary predictors [16]. However, C-statistics are not sufficiently sensitive to assess the effect of important new predictors in risk prediction models [17], thus inaccurately reflecting the real effect of dietary predictors in noninvasive diabetes risk models. To solve this dilemma, net reclassification improvement (NRI) and integrated discrimination improvement (IDI) have been proposed as better measures of discrimination than C-statistics when evaluating new predictors [18]. Based on these measures, a previous study has demonstrated that dietary information is useful in cardiovascular risk models [19]. To date, no study has explicitly evaluated the effect of dietary predictors in diabetes risk models employing these measures.

Therefore, the primary purpose of this study was to develop a noninvasive diabetes risk model that included dietary predictors based on baseline data from a cohort of 9,734 subjects in China and to evaluate the risk model in another independent cohort for external validation. Additionally, we evaluated whether dietary predictors improved model performance and the predictive ability of the noninvasive diabetes risk model.

## Materials and Methods

### The derivation data

In our study, baseline data for the Harbin Cohort Study on Diet, Nutrition and Chronic Noncommunicable Disease (HDNNCDS) were used as the derivation data. The HDNNCDS was launched in 2010 by the national key discipline, Department of Nutrition and Food Hygiene at Harbin Medical University [20]. The HDNNCDS covered 7 urban administrate regions of Harbin. Each region was divided into 3 strata according to their financial situation and a total of 42 communities were randomly selected from each stratum in each administrate region by performing a stratified multistage random cluster sampling design. Subjects were eligible to participate in the study if they 1) were between 20 and 74 years old, 2) had been living in Harbin for at least two years, and 3) were without cancer or type 1 diabetes mellitus. The diabetes diagnosis records were used to distinguish the participants’ diabetes type. A total of 9,734 subjects participated in the HDNNCDS. The first follow-up survey of all cohort subjects in the HDNNCDS is ongoing and is not yet completed. In our analysis, we excluded subjects at baseline who had undergone dietary intervention for diabetes or other diseases (n = 655) and had a total calorie intake ≥ 4500 kcal/d or ≤ 500 kcal/d (n = 315). A total of 8,764 subjects including 1,096 individuals with diabetes were included for analysis in the HDNNCDS.

### The validation data

The cohort data from the Harbin People’s Health Study (HPHS) was used as the validation data. The HPHS was launched in 2008 by the Centers for Disease Control and Prevention and the Public Health School in Harbin [21]. The HPHS covered 5 urban administrate regions of Harbin. Each region were divided into 3 strata based on their financial situation and one or two neighbourhood committees were chosen from 15 communities that were randomly selected from each stratum in each administrate region by performing a stratified multistage random cluster sampling design. A total of 8,940 subjects, aged 20 to 74 years old, were recruited for the study. Subjects were eligible to participate in the study if they had no history of using postmenopausal hormone therapy, malignancy, thyroid dysfunction, renal calculi, corticosteroid or calcitriol use. A total of 4,515 subjects (approximately 50.5% of the total subjects) were randomly selected to participate in the follow-up survey due to the study's financial constraints. In 2012, 4,158 subjects completed the first in-person follow-up survey with a response rate of 92.1%. In the validation data, subjects were also excluded at baseline in HPHS if they had diabetes (n = 550), were undergoing dietary interventions for diabetes or other diseases (n = 126), and had a total calorie intake ≥ 4500 kcal/d or ≤ 500 kcal/d (n = 52), leaving 3,430 subjects included in the analysis. In total, 394 incident cases of type 2 diabetes were observed during the 4.2 years of follow-up.

### Ethics statement

Both of these two study protocols were approved by the Ethics Committee of the Harbin Medical University, and written informed consent was provided by all subjects. The methods in this study were in accordance with the approved guidelines.

### Questionnaire survey

Detailed in-person interviews were administered by trained personnel using a structured questionnaire to collect information on demographic characteristics, dietary habits, lifestyles, physical condition and anthropometric characteristic. The questions about dietary habits, lifestyles and physical condition in the questionnaire were the same in the HDNNCDS and the HPHS.

The section on dietary habits was evaluated by the validated food frequency questionnaire (FFQ) containing data regarding usual dietary intake over the past 12 months, including 103 food items from 14 food groups, which were white rice, wheaten food, potato and its products, beans and its products, fresh vegetables, fresh fruits, livestock and its products, poultry and its products, milk and dairy products, eggs and its products, fish and its products, snack, beverage and ice cream. For each food item, participants are asked to choose their usual rate of consumption frequency categories from “per day”, “per week”, “per month”, and “never” and then answered the number of times for the corresponding frequency categories. The question regarding the amount of food intake consumed in lians (a unit of weight equal to 50 g) or ml (for liquid food item) for the corresponding frequency was measured by using molds of photographs of standard potion sizes. Each food items were quantified in g/d with multiplying the frequency by the amount of the food item. The energy intake per day was estimated by the Food Nutrition Calculator (V1.60, Chinese Center for Disease Control [CDC], Beijing, China).

The section on lifestyles and physical condition mainly included prior disease history, a family history of disease, regular exercise, labor intensity, cigarette smoking, alcohol consumption, and taking medicines and health products in the prior 12 months. Current smokers were defined as those who smoked at least 100 cigarettes in a lifetime or smoked every day or currently smoked some days. Current drinkers were defined as those who consumed ≥ 1 alcoholic drink each month in the 12 months prior to the survey. Regular exercise was defined as any kind of recreational or sport physical activity other than walking for work or life performed at least 30 minutes for three or more days per week.

### Anthropometric measurements and biochemical analyses

Anthropometric measurements, including height, weight, and waist circumference, were also taken at baseline by well-trained examiners, with subjects wearing light, thin clothing and no shoes. Body weight and height were measured to the nearest 0.1 kg and 0.1 cm, respectively. Blood pressures were measured 3 times with a standard mercury sphygmomanometer on the right arm of each subject after a 10-minute rest in a sitting position, and the mean values were used for analysis. Body mass index (BMI) was calculated as weight (kg) divided by the square of the height in meters (m^2^). An oral glucose tolerance test was administered according to the World Health Organization guidelines for each subject. Fasting and 2-h postprandial serum blood glucose levels were measured using an automatic biochemistry analyzer (Hitachi, Japan).

### Outcome ascertainment

Diabetes was identified by self-reports of a history of a diagnosis of diabetes, fasting blood glucose ≥ 7.0 mmol/L, and/or 2-h glucose ≥ 11.1 mmol/L, and/or taking medication for diabetes.

Hypertension was identified by self-reports of a history of a diagnosis of hypertension, a systolic blood pressure ≥ 140 mmHg and/or diastolic blood pressure ≥ 90 mmHg, and/or taking medication for hypertension.

### Statistical analysis

All statistical analyses were performed using SPSS v21.0 (Beijing Stats Data CO. Ltd, Beijing China) and R 2.15.1 (http://www.r-project.org/). A two-sided *P*<0.05 was considered statistically significant.

Student’s t-test and the χ^2^ test were used for the comparison to baseline clinical characteristics and dietary information between the derivation data and validation data. In this study, noninvasive risk factors were defined as factors that could be measured without taking a blood sample. The variables included in the model were divided into classic risk factors and dietary predictors. Classic risk factors included age, gender, BMI, waist circumference, regular exercise, labor intensity, alcohol consumption, cigarette smoking, education, hypertension and a family history of diabetes. Dietary predictors included the 14 food groups in the FFQ and total calorie intake. Their adjusted odds ratio (OR) and 95% confidence intervals (95% CI) were estimated using a multivariate backward logistic regression analysis. The diabetes risk score was developed for the two multivariate logistic regression models: a classic noninvasive risk score and a diet-containing risk score, and points were assigned to each variable based on the magnitude of its regression coefficient [22]. In the diabetes risk score system, we transformed the unit of diet consumption from gram per day to liang per day, to facilitate its use in China. The dietary predictors except diary in the risk model were continuous in order to use the full information for dietary predictors. The risk score allows decimal value if the weight is not in round number. For the dairy products, because 24.4% and 29.4% of the total subjects in the derivation data and validation data were never consume milk and its products, we transformed the dairy and its products variable into binary variable in the risk model. A total diabetes risk score for each individual was calculated as the sum of the points for each variable.

The classic noninvasive risk model and the diet-containing risk model were compared with Akaike’s information criterion (AIC), area under curve (AUC), IDI, NRI and the Hosmer-Lemeshow goodness-of-fit test (HL test) for model performance, discrimination and predictive ability, and general calibration in derivation data and validation data separately. An AIC difference between two models of 10 or greater was considered to be significant with a lower AIC value indicating a better model performance [23]. AUC, IDI and NRI were calculated to evaluate the model discrimination and predictive ability [24]. To apply NRI in the logistic regression model, we adopted the approach proposed by Pencina et al [25]. The Hosmer-Lemeshow goodness-of-fit test (HL test) was used to examine how well the predicted prevalence matched the observed prevalence of type 2 diabetes, and *P*-values of 0.01 or greater from the test were considered to indicate a good calibration.

## Results

Table 1 shows the baseline clinical characteristics and average daily diet intakes in the derivation and validation data. The derivation data were older than the validation data, and the proportion of males was higher in the derivation data than in the validation data. The other baseline characteristics and average daily diet intakes were very different between the derivation and validation data.

**Table 1:** Characteristics of Subjects in the Derivation and Validation Data.

	Derivation data (8,764)	Validation data (3,430)	*P*-value
Age (years)	49.6 (10.3)	44.7 (10.3)	<0.001
Male, n (%)	3,065 (35.0)	1,021 (29.8)	<0.001
Body mass index (kg/m^2^)	24.8 (3.4)	25.0 (3.4)	0.020
Waist circumference (cm)	83.9 (9.9)	85.4 (10.1)	<0.001
Current smokers, n (%)	1,513 (17.4)	544 (15.9)	0.021
Current alcohol drinkers, n (%)	3,191 (36.4)	1,014 (29.6)	<0.001
Hypertension diagnosed, n (%)	3,048 (34.8)	1,158 (33.8)	0.149
Family history of diabetes, n (%)	1,285 (14.7)	417 (12.2)	<0.001
Regular exercise, n (%)	3,967 (45.3)	1,912 (55.7)	<0.001
Fasting serum glucose (mmol/l)	4.91 (1.63)	4.70 (0.71)	<0.001
Postprandial serum glucose (mmol/l)	6.29 (2.87)	5.67 (1.67)	<0.001
***Average daily diet intakes***			
Total calorie (kcal/d)	2,391 (892)	2,253 (845)	<0.001
White rice (g/d)	221 (155)	207 (148)	<0.001
Wheaten food (g/d)	135 (109)	138 (106)	0.180
Potato and its products (g/d)	63 (77)	59 (68)	0.040
Beans and its products (g/d)	53 (66)	50 (61)	0.010
Fresh Vegetable (g/d)	268 (216)	294 (231)	<0.001
Fresh Fruit (g/d)	152 (154)	164 (177)	<0.001
livestock and its products (g/d)	78 (85)	65 (70)	<0.001
Poultry and its products (g/d)	32 (54)	23 (37)	<0.001
Dairy and its products (ml/d)	90 (105)	93 (109)	0.194
Eggs (g/d)	46 (47)	41 (40)	<0.001
Fish and its products (g/d)	37 (79)	33 (80)	0.021
Snacks (g/d)	15 (34)	13 (29)	0.002
Beverage (ml/d)	39 (89)	34 (88)	0.012
Ice-cream (g/d)	11 (29)	9.0 (29)	0.010

All values are presented as the mean (standard derivation) or as percentages.

Student’s t-test and the χ^2^ test were used for the comparison to baseline clinical characteristics and average daily diet intakes between the derivation data and validation data.

Table 2 presents the classic noninvasive risk model and the diet-containing risk model in the derivation data. In the classic noninvasive risk model, age, gender, hypertension, a family history of diabetes, alcohol consumption, regular exercise, BMI and waist circumference were significantly associated with the risk of type 2 diabetes. In the diet-containing risk model, the classic risk factors and consumption of staple foods, livestock, eggs, potato, dairy and its products, fresh fruit and vegetable were retained in the model. The detailed risk score systems based on the two models were presented in the S1 Table.

**Table 2:** Risk Scores Based on the Classic Noninvasive Risk Model and the Diet-containing Risk Model for Type 2 Diabetes Risk in the derivation Data.

	Classic noninvasive risk model			Diet containing noninvasive risk model		
	β	OR (95% CI)	Points allocate	β	OR (95% CI)	Points allocated
***Classic noninvasive risk factors***						
Age (years)	0.060	1.06 (1.05–1.07)	0.6	0.064	1.07 (1.06–1.08)	0.6
Gender, female	−0.597	0.55 (0.47–0.65)	−6	−0.408	0.66 (0.56–0.79)	−4
Hypertension *	0.779	1.92 (1.66–2.22)	8	0.772	2.16 (1.87–2.51)	8
Family history of diabetes †	1.318	3.00 (2.53–3.55)	13	1.394	4.03 (3.38–4.82)	14
Alcohol consumption	0.392	1.48 (1.26–1.75)	4	0.463	1.56 (1.34–1.88)	5
Regular exercise ‡	−0.172	0.84 (0.73–0.96)	−2	−0.233	0.79 (0.69–0.91)	−2
Body mass index (kg/m^2^)	0.063	1.07 (1.04–1.10)	0.6	0.066	1.07 (1.04–1.10)	0.7
Waist circumference (cm)	0.031	1.03 (1.02–1.04)	0.3	0.030	1.03 (1.02–1.04)	0.3
***Dietary factors***						
Staple foods (liang/d) §,**				0.080	1.08 (1.05–1.11)	0.8
Livestock and its products (liang/d)				0.111	1.12 (1.09–1.15)	1
Eggs (liang/d)				0.115	1.12 (1.05–1.20)	1
Potato and its products (liang/d)				−0.147	0.86 (0.82–-0.91)	-1
Fresh fruit (liang/d)				−0.123	0.88 (0.86–0.91)	-1
Fresh vegetable (liang/d)				−0.048	0.95 (0.94–0.97)	-0.5
Dairy and its products #				−0.228	0.80 (0.68–0.94)	-2
C-statistics	0.782 (0.767–0.795)			0.805 (0.791–0.817)		

* Hypertension was defined as self-reports of a history of a diagnosis of hypertension, a systolic blood pressure ≥ 140 mmHg and/or diastolic blood pressure ≥ 90 mmHg, and/or taking medication for hypertension.

† Family history of diabetes was defined as diabetes in first- or second-degree relatives.

‡ Regular exercise was defined as any kind of recreational or sport physical activity other than walking for work or life performed at least 30 minutes for three or more days per week.

§ Consumption of staple foods was defined as the sum of the consumption of white rice and wheaten food.

# Dairy and its products were defined as daily consumption of dairy products.

** 1 Chinese liang ≈ 50 gram

The detailed parameters of the risk model evaluation that dietary predictors improved in the derivation data are presented in Table 3. Dietary predictors improved the model performance with a significantly decreased AIC value (delta AIC = 199.5). Dietary predictors improved the model discrimination with a significant increase in the AUC (delta AUC = 0.03, *P*<0.001). Compared with the classic noninvasive risk model, the addition of dietary predictors to the risk model significantly increased the relative IDI and NRI by 24.6% and 0.155 separately. Based on the results of the HL-test, the predicted prevalence of type 2 diabetes in the two models matched the observed prevalence well (χ^2^ = 7.29; *P* = 0.51 for the classic noninvasive risk model; χ^2^ = 6.34, *P* = 0.61 for the diet-containing risk model). The sensitivity of the diet-containing risk model in the derivation data was significantly higher than that of classic noninvasive risk model at the 20% probability cut-off point (57.6% vs 50.3%, *P*<0.001). The specificity of the two models did not differ significantly in the derivation data (84.5% vs 84.7%, P = 0.389).

**Table 3:** The Detailed Parameters of the Risk Model Evaluation that Dietary Predictors Improved in the Derivation Data.

	Classic noninvasive diabetes risk model	Diet-containing noninvasive diabetes risk model
AIC	5565.3	5365.8
AUC (95% CI)	0.78 (0.77–0.80)**	0.81 (0.79–0.82)**
*P*-value for difference in C-statistics	Reference	<0.001
IDI (95% CI)	Reference	0.033 (0.028–0.039)**
Relative IDI	Reference	24.6%
NRI (95% CI)	Reference	0.155 (0.120–0.190)**
HL χ^2^ (*P*-value)	7.29(0.51)	6.34(0.61)

AIC, Akaike’s information criterion; AUC, area under curve; HL, Hosmer-Lemeshow goodness-of-fit; IDI, integrated discrimination improvement, NRI, net classification improvement

** *P*<0.001.

**Fig 1:**
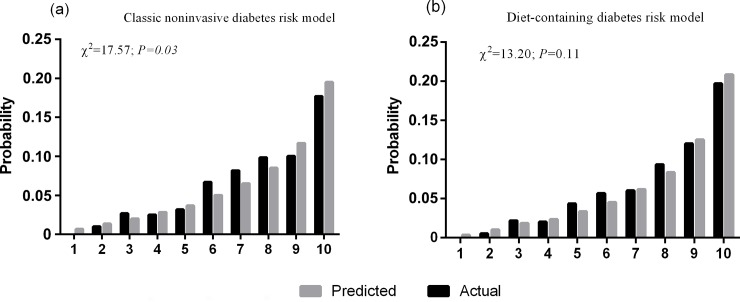
The Hosmer-Lemeshow Goodness-of-fit Test for the Diet-containing Diabetes Risk Model and Classic Noninvasive Diabetes Risk Model. X-axes indicate the deciles of the predicted risk of type 2 diabetes, and y-axes indicate the probability of type 2 diabetes events. *P*-values from χ^2^ statistics calculated to compare the difference between the predicted and the actual incidence of type 2 diabetes.

**Fig 2:**
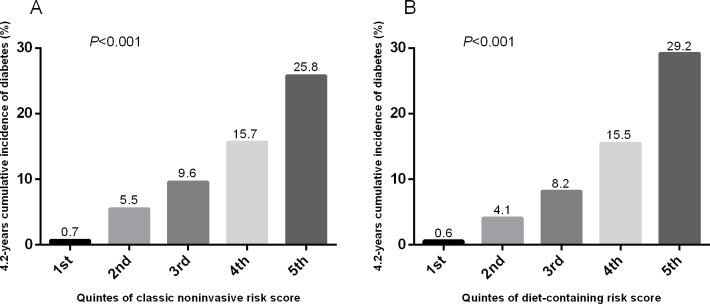
**Estimated 4.2 years cumulative incidence of type 2 diabetes by quintile of risk scores of the classic noninvasive risk model (A) and diet-containing risk model (B).** X-axes indicate the quintiles of the two risk scores, and y-axes indicate the 4.2 years cumulative incidence of type 2 diabetes. *P*-values from χ^2^ statistics calculated to compare the difference of the incidence of type 2 diabetes across the quintiles of the two risk scores.

To further validate the usefulness of the diet-containing risk model of the derivation data and to evaluate the effect of dietary predictors on the diabetes risk model, the scoring methods were ascertained by applying the scores to the validation data. The distribution of the scores in both models of both dataset was normal distribution by K-S test (classic noninvasive risk score in the derivation data: Z = 1.25, *P* = 0.090; in the validation data: Z = 0.953, *P* = 0.323; diet-containing risk score in the derivation data: Z = 1.318, *P* = 0.062; in the validation data: Z = 0.812, *P* = 0.525). The score distribution of the classic noninvasive risk model in the derivation data (median 68.5, standard deviation 11.8, range from 21.7 to 117.9) was not significantly different from that in the validation data (median 68.2, standard deviation 11.4, range from 34.1 to 107.8) (*P* = 0.060), whereas the score distribution of the diet-containing risk model was shifted significantly to higher levels in the validation data (median 72.8, standard deviation 13.0, range from 20.6 to 127.7) compared with the derivation data (median 71.5, standard deviation 12.4, range from 30.4 to 112.1) (*P*<0.001). As shown in Table 4, in the validation data, the sum of the scores of the diet-containing risk model predicted the incidence of type 2 diabetes more effectively than the sum of the scores based on the classic risk model, with a significant increase in the AUC (delta AUC = 0.03, *P*<0.001). Compared with the classic noninvasive risk model, the addition of dietary predictors to the model significantly increased the relative IDI and NRI by 22.5% and 0.219 separately. Based on the results of the HL-test, the calibration of the diet-containing risk model was better than that of the classic noninvasive risk model (χ^2^ = 13.20; *P* = 0.11 for the diet-containing risk model; χ^2^ = 17.57, *P* = 0.03 for the classic noninvasive risk model). Detailed information for the HL-test in the two models is presented in Fig 1. The 4.2-years cumulative incidence of type 2 diabetes was significantly increased with elevating quintiles of the risk scores of both models as shown in the Fig 2. The sensitivity of the diet-containing risk model in the validation data was significantly higher than that of classic noninvasive risk model at the 20% probability cut-off point (49.0% vs 40.4%, *P*<0.001). The specificity of the two models did not differ significantly (85.8% vs 85.7%, *P* = 0.543).

**Table 4:** The Detailed Parameters of the Risk Model Evaluation that Dietary Predictors Improved in the Validation Data.

	Classic noninvasive risk model	Diet-containing noninvasive risk model
AIC	2116.6	1934.5
AUC (95% CI)	0.76 (0.73–0.78)**	0.79 (0.77–0.81)**
*P*-value for difference in C-statistics	Reference	<0.001
IDI (95% CI)	Reference	0.026 (0.018–0.033)**
Relative IDI	Reference	22.5%
NRI (95% CI)	Reference	0.219 (0.158–0.281)**
HL χ^2^ (*P*-value)	17.57 (0.03)	13.20 (0.11)

AIC, Akaike’s information criterion; AUC, area under curve; HL, Hosmer-Lemeshow goodness-of-fit; IDI, integrated discrimination improvement, NRI, net classification improvement

** *P*<0.001.

## Discussion

In the present study, we developed a diet-containing noninvasive type 2 diabetes risk model for Northern Chinese using dietary information and classic noninvasive risk factors. We observed that the addition of dietary predictors to the noninvasive diabetes risk model improved the model performance and its predictive ability.

Many noninvasive risk models have been developed to predict the risk of type 2 diabetes [1–11]. Some of them have been developed for Asians [6–11], which also can be adapted to the setting and purpose of our study. Most of these risk models included the following classic risk factors: age, gender, BMI, WC, family history of diabetes, hypertension, sport time, smoking and alcohol consumption. We also included all of these risk factors in the classic noninvasive risk model in our study, and the effects of these risk factors were in agreement with previous studies, which could be seen as the update and utility of these risk models in our population. Therefore, the performance of the classic noninvasive risk model in our study can represent the performance of these previous risk models, and dietary information will improve the model performance and predictive ability of these previous risk models.

The effect of diet has been widely studied and acknowledged. For example, a prospective study showed that the consumption of milk and dairy products is associated with a markedly reduced prevalence of the metabolic syndrome [26]. A meta-analysis also suggested that 1.35 servings a day of vegetables compared with 0.2 servings resulted in a 14% reduction of the risk of type 2 diabetes [27], a 6% lower risk of type 2 diabetes per 1 serving a day increment of fruit intake [28], and an 11% increase in the risk of type 2 diabetes per 1 serving a day of white rice consumption [29]. However, dietary predictors have not been adequately emphasized in type 2 diabetes risk prediction models. Simmons et al. reported that the addition of dietary predictors does not improve the performance of the model or the predictive ability of type 2 diabetes risk models. The authors speculated that risk factors such as BMI interfere with the effects of long-term diet and future diabetes risks, and this may account for the lack of improvement with the addition of dietary predictors to the model. However, these classic risk factors cannot completely account for the association between diet and type 2 diabetes. For example, a previous study showed that increased red meat consumption over time was associated with an elevated subsequent risk of type 2 diabetes, and the association was only partially mediated by body weight [30]. In addition, the value of C-statistics is insensitive when evaluating new predictors. In our study, we used new measures such as NRI and IDI to evaluate the effects of dietary predictors in the noninvasive diabetes risk model. We included 14 kinds of food that are common in China. We found potato, dairy products, fresh fruit and vegetables decreased the risk of type 2 diabetes, and staple foods including white rice and wheaten food, eggs and livestock increased the risk, which is consistent with previous studies. In the present study, the diet-containing risk model had better AIC and AUC than the classic noninvasive risk model in the derivation and validation data. The addition of dietary predictors to the risk model yielded a significant increase in relative IDI and NRI for the risk model compared with the classic noninvasive risk model. Furthermore, the Hosmer-Lemeshow goodness-of-fit test for the diet-containing noninvasive risk model indicated better agreement between the observed and predicted probabilities of type 2 diabetes incidence across deciles than that of the classic noninvasive risk model in the validation data. The increased sensitivity also indicated that the diet-containing risk model identified 7 and 8 more patients than classic noninvasive risk model per one hundred diabetic patients in the derivation data and validation data. These results indicate that dietary predictors can improve the model performance and predictive ability of the noninvasive diabetes risk model. Dietary information is useful for constructing a noninvasive type 2 diabetes risk model.

In China, currently more than half of the people with diabetes are undiagnosed [31], and the burden of expenditures for medication and care is rising as the incidence of type 2 diabetes increases rapidly. Although the existing classic noninvasive diabetes risk models may be helpful for identifying high risk subjects in the primary care settings, they are probably not efficient tools for preventing diabetes in clinical practice for the reason that they include limited modifiable risk factors. The diet-containing noninvasive risk model can improve disease awareness and identify high-risk individuals and potential targets for the reduction of type 2 diabetes risk. Most of the risk factors in the diet-containing risk model are modifiable. Therefore, this model can establish specific, effective and feasible strategies for the prevention of type 2 diabetes and motivate patients to comply with a healthy lifestyle and with treatment plans. The dietary information in our study came from FFQ. The FFQ in our study presents 103 food items, and it takes 20–30 minutes to finish and can self-administered. This method enables to obtain dietary data in a relative simple, cost-effective and time efficient approach. It is suitable and feasible for the integration of FFQ into noninvasive diabetes risk models. Although dietary record provides more accurate dietary data of an individual for diet evaluation than FFQ does in a clinical setting, time and labor intensive are two major constraints of dietary record, which have been suggested as barriers for physicians to apply this method in clinical practice.

The strength of our study is that we included dietary predictors in the type 2 diabetes risk prediction model, providing more modifiable factors than previous studies in the noninvasive risk model for the Asian population. Furthermore, we validated the usefulness of dietary predictors in the noninvasive type 2 diabetes risk model in the derivation data and in another independent cohort. Our studies also have limitations. First, we cannot exclude the possibility of measurement errors from information bias in the dietary data, although trained personnel performed the interviews using a validated FFQ and molds and photos of portion sizes. The reproducibility and validity of the FFQ in our study has been assessed in the previous studies, and the results indicated that the FFQ in our study is a reliable method for assessing dietary intake [20, 21]. Therefore, the measurement errors from information bias due to food data may affect the predictive ability very slightly. Additionally, our analysis did not include a section on nutrient supplementation, which has been found to be associated with type 2 diabetes and may lead to potential biases. Second, the two risk models were developed based on cross-sectional data because of its large sample size and relative good representation of the area. Although the two risk models in our study could only associate prevalent cases of type 2 diabetes, rather than identify incident cases, the discrimination and predictive ability of the diet-containing risk model were relative good with higher sensitivity and acceptable AUC when evaluated it in an independent cohort.

## Conclusion

In conclusion, we developed a diet-containing noninvasive risk model for a northern Chinese population using classic noninvasive risk factors and dietary information. Dietary information improves model performance and predictive ability of noninvasive type 2 diabetes risk model based on classic risk factors. Dietary information may be useful for developing noninvasive type 2 diabetes risk models.

## Supporting Information

S1 Table The detailed risk score systems based on the two models. (DOCX)

## References

[1] Baan CA, Ruige JB, Stolk RP, Witteman JC, Dekker JM, Heine RJ, et al Performance of a predictive model to identify undiagnosed diabetes in a health care setting. Diabetes Care. 1999;22: 213–219. 10.2337/diacare.22.2.213 10333936

[2] American Diabetes Association. Screening for type 2 diabetes. Diabetes Care. 1998;21 (Suppl 1): S20–S23. 10.2337/diacare.21.1.S20 12017671

[3] Lindström J, Tuomilehto J. The diabetes risk score: a practical tool to predict type 2 diabetes risk. Diabetes Care. 2003;26: 725–731. 10.2337/diacare.26.3.725 12610029

[4] Glümer C, Carstensen B, Sandbaek A, Lauritzen T, Jørgensen T, Borch-Johnsen K. A Danish diabetes risk score for targeted screening: the Inter99 study. Diabetes Care. 2004;27: 727–733. 10.2337/diacare.27.3.727 14988293

[5] Schulze MB, Hoffmann K, Boeing H, Linseisen J, Rohrmann S, Mohlig M, et al An accurate risk score based on anthropometric, dietary, and lifestyle factors to predict the development of type 2 diabetes. Diabetes Care. 2007;30: 510–515. 10.2337/dc06-2089 17327313

[6] Aekplakorn W, Bunnag P, Woodward M, Sritara P, Cheepudomwit S, Yamwong S, et al A risk score for predicting incident diabetes in the Thai population. Diabetes Care. 2006;29: 1872–1877. 10.2337/dc05-2141 16873795

[7] Gao WG, Dong YH, Pang ZC, Nan HR, Wang SJ, Ren J, et al A simple Chinese risk score for undiagnosed diabetes. Diabet Med. 2010;27: 274–281. 10.1111/j.1464-5491.2010.02943.x 20536489

[8] Doi Y, Ninomiya T, Hata J, Hirakawa Y, Mukai N, Iwase M, et al Two risk score models for predicting incident type 2 diabetes in Japan. Diabet Med. 2012;29: 107–114. 10.1111/j.1464-5491.2011.03376.x 21718358

[9] Sun F, Tao Q, Zhan S. An accurate risk score for estimation 5-year risk of type 2 diabetes based on a health screening population in Taiwan. Diabetes Res Clin Pract. 2009; 85(2): 228–34. 10.1016/j.diabres.2009.05.005 19500871

[10] Lee YH, Bang H, Kim HC, Kim HM, Park SW, Kim DJ. A simple screening score for diabetes for the Korean population: development, validation, and comparison with other scores. Diabetes Care 2012; 35: 1723–1730. 10.2337/dc11-2347 22688547 PMC3402268

[11] Zhou X, Qiao Q, Ji L, Ning F, Yang W, Weng J, et al Nonlaboratory-based risk assessment algorithm for undiagnosed type 2 diabetes developed on a nation-wide diabetes survey. Diabetes Care 2013;36: 3944–3952. 10.2337/dc13-0593 24144651 PMC3836161

[12] Glümer C, Vistisen D, Borch-Johnsen K, Colagiuri S, DETECT-2 Collaboration. Risk scores for type 2 diabetes can be applied in some populations but not all. Diabetes Care. 2006;29: 410–414. 10.2337/diacare.29.02.06.dc05-0945 16443896

[13] Tuomilehto J, Lindström J, Eriksson JG, Valle TT, Hämäläinen H, Ilanne-Parikka P, et al Prevention of type 2 diabetes mellitus by changes in lifestyle among subjects with impaired glucose tolerance. N Engl J Med. 2001;344: 1343–1350. 10.1056/NEJM200105033441801 11333990

[14] Knowler WC, Barrett-Connor E, Fowler SE, Hamman RF, Lachin JM, Walker EA, et al Reduction in the incidence of type 2 diabetes with lifestyle intervention or metformin. N Engl J Med. 2002;346: 393–403. 10.1056/NEJMoa012512 11832527 PMC1370926

[15] Lindström J, Ilanne-Parikka P, Peltonen M, Aunola S, Eriksson JG, Hemiö K, et al Sustained reduction in the incidence of type 2 diabetes by lifestyle intervention: follow-up of the Finnish diabetes prevention study. Lancet. 2006;368: 1673–1679. 10.1016/S0140-6736(06)69701-8 17098085

[16] Simmons RK, Harding AH, Wareham NJ, Griffin SJ, EPIC-Norfolk Project Team. Do simple questions about diet and physical activity help to identify those at risk of type 2 diabetes? Diabet Med. 2007;24: 830–835. 10.1111/j.1464-5491.2007.02173.x 17490419

[17] Cook NR. Use and misuse of the receiver operating characteristic curve in risk prediction. Circulation. 2007;115: 928–935. 10.1161/CIRCULATIONAHA.106.672402 17309939

[18] McGeechan K, Macaskill P, Irwig L, Bossuyt PM. An assessment of the relationship between clinical utility and predictive ability measures and the impact of mean risk in the population. BMC Med Res Methodol. 2014;14: 86–98. 10.1186/1471-2288-14-86 24989719 PMC4105158

[19] Baik I, Cho NH, Kim SH, Shin C. Dietary information improves cardiovascular disease risk prediction models. Eur J Clin Nutr. 2013;67: 25–30. 10.1038/ejcn.2012.175 23149979

[20] Na L, Wu X, Feng R, Li J, Han T, Lin L, et al The Harbin cohort study on diet, nutrition and chronic non-communicable diseases: study design and baseline characteristics. PLOS ONE. 2015;10: e0122598 10.1371/journal.pone.0122598 25856294 PMC4391912

[21] Huang L, Xue J, He Y, Wang J, Sun C, Feng R, et al Dietary calcium but not elemental calcium from supplements is associated with body composition and obesity in Chinese women. PLOS ONE. 2011;6: e27703 10.1371/journal.pone.0027703 22163269 PMC3233543

[22] Sullivan LM, Massaro JM, D'Agostino RB. Presentation of multivariate data for clinical use: The Framingham Study risk score functions. Stat Med 2004;23: 1631–1660. 10.1002/sim.1742 15122742

[23] Koenig W, Löwel H, Baumert J, Meisinger C. C-reactive protein modulates risk prediction based on the Framingham score: implications for future risk assessment: results from a large cohort study in southern Germany. Circulation. 2004;109: 1349–1353. 10.1161/01.CIR.0000120707.98922.E3 15023871

[24] Steyerberg EW, Vickers AJ, Cook NR, Gerds T, Gonen M, Obuchowski N, et al Assessing the performance of prediction models: a framework for traditional and novel measures. Epidemiology. 2010;21: 128–138. 10.1097/EDE.0b013e3181c30fb2 20010215 PMC3575184

[25] Pencina MJ, D'Agostino RB, Steyerberg EW. Extensions of net reclassification improvement calculations to measure usefulness of new biomarkers. Stat Med. 2011;30: 11–21. 10.1002/sim.4085 21204120 PMC3341973

[26] Elwood PC, Pickering JE, Fehily AM. Milk and dairy consumption, diabetes and the metabolic syndrome: the Caerphilly prospective study. J Epidemiol Comm Health. 2007;61: 695–698. 10.1136/jech.2006.053157 17630368 PMC2652996

[27] Carter P, Gray LJ, Troughton J, Khunti K, Davies MJ. Fruit and vegetable intake and incidence of type 2 diabetes mellitus: systematic review and meta-analysis. BMJ. 2010;341: c4229 10.1136/bmj.c4229 20724400 PMC2924474

[28] Li M, Fan Y, Zhang X, Hou W, Tang Z. Fruit and vegetable intake and risk of type 2 diabetes mellitus: meta-analysis of prospective cohort studies. BMJ Open. 2014;4: e005497 10.1136/bmjopen-2014-005497 25377009 PMC4225228

[29] Hu EA, Pan A, Malik V, Sun Q. White rice consumption and risk of type 2 diabetes: meta-analysis and systematic review. BMJ. 2012;344: e1454 10.1136/bmj.e1454 22422870 PMC3307808

[30] Pan A, Sun Q, Bernstein AM, Manson JE, Willett WC, Hu FB. Changes in red meat consumption and subsequent risk of type 2 diabetes mellitus: three cohorts of US men and women. JAMA Intern Med. 2013;173: 1328–1335. 10.1001/jamainternmed.2013.6633 23779232 PMC3847817

[31] Yang SH, Dou KF, Song WJ. Prevalence of diabetes among men and women in China. N Engl J Med. 2010;362: 2425–2426; [Author reply: 2426]. doi: 10.1056/NEJMc1004671 20578276

