# Supplementary material for: Dietary Information Improves Model Performance and Predictive Ability of a Noninvasive Type 2 Diabetes Risk Model
Source: PLoS One. 2016 Nov 16;11(11):e0166206. doi: 10.1371/journal.pone.0166206 (PMC5112856; doi:10.1371/journal.pone.0166206)
Supplement: S1 Table — (DOCX) [file pone.0166206.s001.docx]

**Supplementary Table 1. The detailed risk score systems based on the two models**

| Risk factors | Classic noninvasive risk score | Diet-containing risk score |
| --- | --- | --- |
| Age (years) | 0.6 | 0.6 |
| Gender  Male=0/Female=1 | −6 | −4 |
| Waist circumference (cm) | 0.3 | 0.7 |
| Body mass index (kg/m^2^) | 0.6 | 0.3 |
| Regular exercise habitus  Yes=0/No=1 | −2 | −2 |
| Alcohol consumption  Yes=1/No=0 | 4 | 5 |
| Hypertension  Yes=1/No=0 | 8 | 8 |
| Family history of diabetes  Yes=1/No=0 | 13 | 14 |
| Intake of staple food (liang/d) |  | 0.8 |
| Intake of livestock and its products (liang/d) |  | 1 |
| Intake of eggs (liang/d) |  | 1 |
| Intake of potato and its products (liang/d) |  | -1 |
| Intake of fresh fruit (liang/d) |  | -1 |
| Intake of fresh vegetable (liang/d) |  | -0.5 |
| Dairy and its products  Yes=1/No=0 |  | -2 |
